# Epistatically Interacting Substitutions Are Enriched during Adaptive Protein Evolution

**DOI:** 10.1371/journal.pgen.1004328

**Published:** 2014-05-08

**Authors:** Lizhi Ian Gong, Jesse D. Bloom

**Affiliations:** Division of Basic Sciences and Computational Biology Program, Fred Hutchinson Cancer Research Center, Seattle, Washington, United States of America; Brown University, United States of America

## Abstract

Most experimental studies of epistasis in evolution have focused on adaptive changes—but adaptation accounts for only a portion of total evolutionary change. Are the patterns of epistasis during adaptation representative of evolution more broadly? We address this question by examining a pair of protein homologs, of which only one is subject to a well-defined pressure for adaptive change. Specifically, we compare the nucleoproteins from human and swine influenza. Human influenza is under continual selection to evade recognition by acquired immune memory, while swine influenza experiences less such selection due to the fact that pigs are less likely to be infected with influenza repeatedly in a lifetime. Mutations in some types of immune epitopes are therefore much more strongly adaptive to human than swine influenza—here we focus on epitopes targeted by human cytotoxic T lymphocytes. The nucleoproteins of human and swine influenza possess nearly identical numbers of such epitopes. However, mutations in these epitopes are fixed significantly more frequently in human than in swine influenza, presumably because these epitope mutations are adaptive only to human influenza. Experimentally, we find that epistatically constrained mutations are fixed only in the adaptively evolving human influenza lineage, where they occur at sites that are enriched in epitopes. Overall, our results demonstrate that epistatically interacting substitutions are enriched during adaptation, suggesting that the prevalence of epistasis is dependent on the underlying evolutionary forces at play.

## Author Summary

Mutations can fix during evolution for two reasons: they can be beneficial and fix for *adaptive* reasons, or they can be neutral or deleterious and fix solely by chance. Most studies focus on adaptation, where the evolving population is increasing in fitness due to a new selection pressure. Such studies have found an important evolutionary role for *epistasis*, the phenomenon where the effect of one mutation depends on another mutation. But adaptation only accounts for a fraction of overall evolutionary change. Here we investigate whether epistasis is as common during non-adaptive as adaptive evolution. We do this by comparing the same protein from human and swine influenza. Human influenza is constantly adapting to escape from the immunity that people acquire from previous influenza infections. But swine influenza is under less pressure to escape from acquired immunity since pigs have shorter lifetimes and are less likely to be infected with influenza multiple times. We find that epistasis is less common during the evolution of the swine influenza protein than its human influenza counterpart. Overall, our results suggest that mutations that interact via epistasis are more likely to fix during adaptive evolution.

## Introduction

Epistasis occurs when the effect of a change at one site in a genome depends on the presence or absence of a change at another site. Understanding epistasis is of profound importance in evolutionary biology, as epistasis can constrain evolutionary pathways and shape patterns of sequence change. As a result, epistasis has been extensively studied at an experimental level. Nearly all of these studies have focused on *adaptive* evolution, where the population is undergoing changes that improve its fitness in response to some new selection pressure. Examples include bacterial adaptation to new environmental conditions [1]–[3], the acquisition of drug resistance [4]–[7], and changes in enzyme activity or specificity [8]–[10]. These studies have almost universally emphasized a crucial role for epistasis in adaptive evolution.

But adaptive evolution accounts for only a portion of total evolutionary change, which can also be driven by stochastic forces such as genetic hitchhiking and drift [11]–[15]. In many cases, these stochastic forces probably drive a greater fraction of overall sequence change than does adaptive evolution [13]–[17]. Do insights about epistasis from studies of adaptive evolution also apply to evolutionary change by non-adaptive forces?

There are reasons to suspect that epistatically interacting substitutions may be more prevalent in adaptive than non-adaptive evolution. Two main mechanisms have been identified for the fixation of epistatically interacting mutations during adaptive evolution: compensatory mutations and permissive mutations. In the compensatory-mutation mechanism, selection favors an initial mutation that confers an overall adaptive benefit but also creates secondary defects, which are then remedied by a subsequent compensatory mutation. An example is the evolution of broad-spectrum antibiotic resistance, where an initial mutation that confers resistance to a new antibiotic but impairs protein stability is followed by a compensatory mutation that restores stability [5], [18], [19]. In this compensatory-mutation mechanism, both epistatic mutations are immediately beneficial.

In the permissive-mutation mechanism, an initially neutral or mildly deleterious [20] mutation that rises in frequency due to stochastic forces is essential for permitting the subsequent than adaptive mutation. An example is the evolution of steroid-receptor specificity, where initial neutral mutations modulate protein conformational stability in a way that permits subsequent adaptive mutations to alter specificity [8]. In this permissive-mutation mechanism, only the subsequent adaptive mutations are directly favored by selection – but selection for the adaptive mutations indirectly favors linked permissive mutations, leading to expansion of lineages carrying the combination of mutations and increasing their rate of fixation [21].

Crucially, in both the compensatory-mutation and the permissive-mutation mechanisms described above, adaptive evolution is ultimately responsible for driving fixation of the epistatic mutations. It is possible to imagine scenarios for the fixation of epistatic mutations by stochastic forces in the absence of adaptation – but it is not immediately obvious whether epistatic mutations would fix as commonly in the absence of a driving selective force. This idea that the frequency of epistatically interacting substitutions might differ between adaptive and non-adaptive evolution would be consistent with theoretical work suggesting that patterns of epistasis depends on the selective forces at play [22], [23].

Here we examine whether epistasis is more common during adaptive evolution by comparing a pair of protein homologs of which only one is subject to a known selection pressure for adaptation. Specifically, we compare nucleoprotein (NP) homologs from human and swine influenza. In both of these influenza lineages, NP has a highly conserved and essential function in the packaging and transcription of viral RNA, and this function is under strong stabilizing selection [24], [25].

Because human influenza circulates in a population of long-lived hosts that are infected with influenza repeatedly during their lifetimes, human influenza is also under constant diversifying selection for adaptive mutations that escape immune memory that accumulates in the host population [26]–[29]. A major way in which human immune memory targets NP is via cytotoxic T lymphocytes (CTLs), and mutations in CTL epitopes are therefore of adaptive value to human influenza [30]–[33]. We have previously shown that the evolution of NP from human influenza involves the fixation of mutations involved in strong epistatic interactions, and that these epistatic mutations occur in epitopes targeted by CTLs [34]. This prior work hints at an association between epistasis and adaptation.

To systematically test the hypothesis that epistasis is enriched during adaptation, here we compare human influenza NP with its swine influenza homolog. Swine influenza is not targeted by human CTLs (CTL epitopes are highly species specific [35], [36]) – so mutations in human CTL epitopes are not of any special significance to swine influenza. Furthermore, swine influenza is unlikely to be under strong diversifying selection even from swine CTLs. In contrast to human influenza, swine influenza circulates in a population of short-lived hosts that have much less opportunity to acquire anti-influenza immune memory before they are slaughtered [37]. As a result, swine influenza is under less pressure to escape from host immune memory. For example, the HA of classical swine influenza underwent minimal antigenic change from 1918 through the late 1990s [37]–[42] – a timeframe during which human influenza HA underwent extremely extensive antigenic change [43], [44]. Although reassortment events and swine vaccination may have recently somewhat increased antigenic change [38]–[40], overall antigenic change in swine influenza is clearly far less than in human influenza [40], [43], [44].

For this reason, the NPs from swine and human influenza represent an ideal pair of homologs for comparative studies of how adaptation affects patterns of epistasis during evolution. While both NPs are under strong stabilizing selection to maintain their essential and conserved biochemical functions [24], [25], only NP from human influenza is under substantial diversifying selection to change sequence epitopes recognized by CTLs. Comparison of the evolution of NPs from these two influenza lineages therefore provides a naturally occurring case study of how ongoing adaptation affects evolutionary patterns.

In the work described below, we first infer evolutionary trajectories for human and swine NP homologs. We then comprehensively mine existing experimental data to define sites in both NP homologs that are targeted by human CTLs. We show that the human NP homolog exhibits an increased frequency of substitutions in these sites relative to the swine NP homolog, a finding consistent with the expectation that mutations to these sites are adaptive only to human influenza. We then experimentally show that the swine NP homolog lacks the type of epistatic mutations that are fixed in the adaptively evolving human NP homolog. Finally, we use our comprehensive analysis of human CTL epitopes to systematically verify that epistatic interactions within the human NP homolog occur at sites that are targeted by CTLs, where mutations are of adaptive value. Overall, these results demonstrate that during NP evolution, epistatically interacting substitutions are enriched during adaptation.

## Results

### Evolutionary trajectories of NP homologs from human and swine influenza

**Figure 1:**
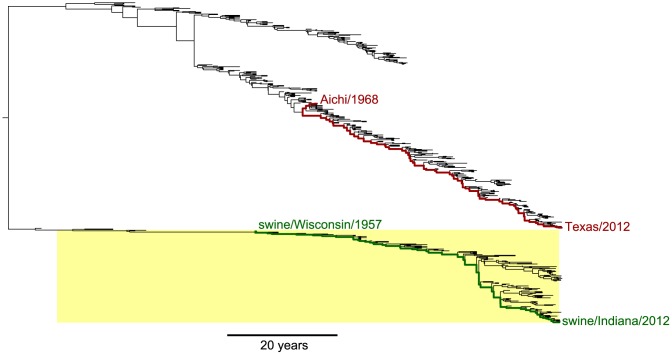
Phylogenetic tree of human and swine NP homologs. The human and swine NP lineages in this tree are descended from a virus closely related to the 1918 virus. Swine viruses are highlighted in yellow; all other viruses are human. In red are the lines of descent to the human H3N2 strains Aichi/1968 and Texas/2012 from their most-recent common ancestor. In green are the lines of descent to the swine H1N1 strains swine/Wisconsin/1957 and swine/Indiana/2012 from their most-recent common ancestor. Overall, this tree shows NPs from the following lineages: human seasonal H1N1, human H2N2, human H3N2, and North American swine viruses. The tree is a maximum clade credibility summary of a posterior distribution sampled from date-stamped protein sequences using BEAST [51] with a JTT [66] substitution model. See http://jbloom.github.io/mutpath/example_influenza_NP_1918_Descended.html for code, input data, and detailed documentation.

We set out to compare the evolution of NP homologs from human and swine influenza. Figure 1 shows a phylogenetic tree of NP from human and swine influenza lineages that derive this gene from a common ancestor closely related to the viruses that caused concurrent human and swine pandemics in 1918 [45], [46]. The NP genes of the human influenza lineages in Figure 1 have circulated exclusively in humans since 1918 [45], [46], while the NP genes of the swine influenza lineages in Figure 1 have circulated exclusively in swine since 1918 [38], [47].

Upon transfer into a new host, influenza undergoes a process of adaptation to the ecology, physiology, cell biology and innate immunology of the new host [48]. Because the details of this host adaptation are incompletely understood, we confined our studies to NP homologs that had already been circulating in their respective hosts for several decades. Our expectation is that during these decades of host-specific evolution, the NP homologs will have become highly adapted to the genetically encoded characteristics of their hosts – and that any further adaptation will be driven largely by non-genetic changes in the hosts, such as the acquisition of immune memory due to prior infections.

We therefore focused on the two evolutionary trajectories indicated in Figure 1. For human influenza, we examined the trajectory separating the H3N2 strains A/Aichi/2/1968 and A/Texas/JMM 49/2012. For swine influenza, we examined the trajectory separating the H1N1 strains A/swine/Wisconsin/1/1957 and A/swine/Indiana/A00968365/2012. In both cases, the starting strains for these trajectories meet the criterion specified in the previous paragraph – they are viruses with NPs that have had several decades to adapt to their respective hosts.

**Figure 2:**
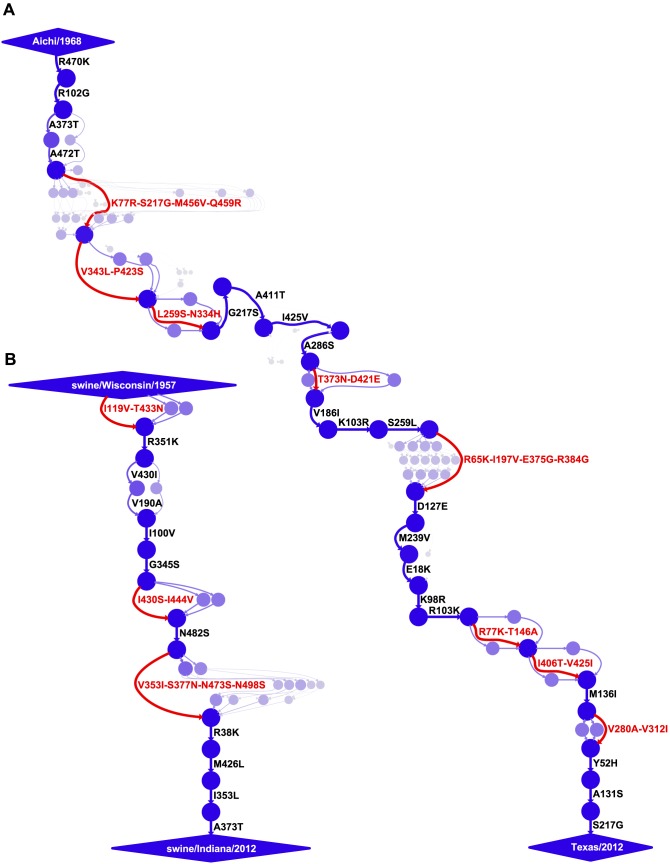
Evolutionary trajectories of human and swine NP. Mutational paths through protein sequence space along (A) the evolutionary trajectory from the human strain Aichi/1968 to Texas/2012 and (B) the evolutionary trajectory from swine/Wisconsin/1957 to swine/Indiana/2012. In the mutational paths, circles represent unique protein sequences, with areas and intensities proportional to the posterior probability that the sequence was part of the trajectory. Blue lines with black labels represent single mutations between sequences, with thicknesses and intensities proportional to the posterior probability that the mutational connection was part of the trajectory. When there is no single high-probability one-mutation connection between sequences, red lines and labels indicate that several mutations fixed in an unknown order. See http://jbloom.github.io/mutpath/example_influenza_NP_1918_Descended.html for code, input data, and detailed documentation. The trajectory in (A) is highly similar to that reported in [34], but is slightly longer and contains sequences from prior to 1968. The inclusion of these pre-1968 sequences is the reason why the first portion of the trajectory is slightly better resolved than that in [34].

In order to map the mutations along these evolutionary trajectories, we utilized a previously described approach [34] for estimating the posterior distribution of mutational paths through protein sequence space by probabilistically placing mutations [49], [50] on trees sampled from a posterior distribution using BEAST [51]. The inferred mutational paths are shown in Figure 2. The human influenza NP accumulated 40 amino-acid mutations along the roughly 44-year trajectory, corresponding to 34 unique mutations relative to the initial Aichi/1968 NP (six mutations are reversions). The swine influenza NP accumulated 18 amino-acid mutations along the roughly 55-year trajectory, corresponding to 18 unique mutations relative to the initial swine/Wisconsin/1957 NP (there are no reversions).

We posit that two factors contribute to the slower rate of amino-acid substitution along the swine NP evolutionary trajectory relative to that of the human NP. First, as discussed in the previous section, the swine NP homolog is under less direct selection from immune memory than its human counterpart. Second, the strongest selection on influenza is from antibodies against the viral surface proteins, and so much of NP's sequence evolution is driven by stochastic genetic hitchhiking with adaptive antibody-escape mutations in these surface proteins [27], [52]. The reduced immune selection on these surface proteins in the swine lineage [37]–[43] probably curtails opportunities for similar genetic hitchhiking by mutations to the swine NP homolog. However, it is important to note that NP function is absolutely essential for viral replication in all strains of influenza [24], [25], and that decreases in NP function dramatically impair viral fitness [34]. Therefore, both adaptive and hitchhiking mutations in NP must first satisfy the stringent stabilizing selection for retention of protein function before they have an opportunity to fix.

### Human and swine NP possess similar numbers of known human CTL epitopes

In order to examine the association between NP evolution and selection from CTLs, we comprehensively mapped human CTL epitopes in the human and swine influenza NP homologs. Numerous experimental studies have identified epitopes in NP that are targeted by human CTLs (see for example [30], [53]–[57] plus many others). The Immune Epitope Database [58] contains a comprehensive listing of such experimentally characterized epitopes. We created a software package (https://github.com/jbloom/epitopefinder) to systematically parse this database for MHC class I epitopes with an experimentally verified human T-cell response that are between 8 and 12 residues in length and align with no more than one mismatch to NP. We considered epitopes to be present in human influenza NP if they matched to either the Aichi/1968 or Texas/2012 NP, and to be present in swine influenza NP if they matched to either the swine/Wisconsin/1957 or swine/Indiana/2012 NP. We removed redundant epitopes from the same MHC class I gene allele group (see http://hla.alleles.org/nomenclature/naming.html) or from the same supertype [59] if the allele group was not specified.

**Figure 3:**
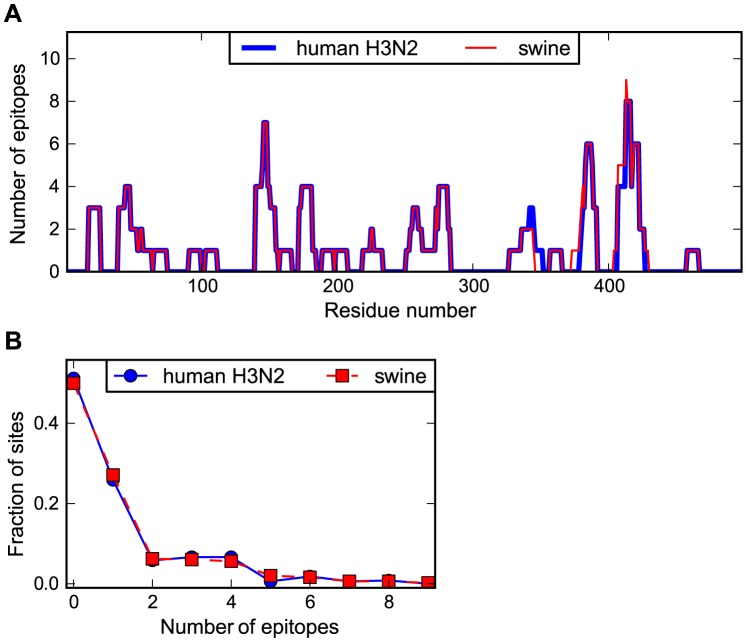
Human and swine NP possess similar numbers of human CTL epitopes. (A) The number of known human CTL epitopes for each residue for human and swine NP. (B) The distribution of number of epitopes per site. The curves in (B) are consistent with the null hypothesis that the human and swine per-site epitope counts are drawn from the same underlying distribution (Kolmogorov-Smirnov test, P = 1.00). The number of epitopes for each site was determined by downloading all human MHC class I epitopes with experimentally verified T-cell responses from the Immune Epitope Database [58], and identifying epitopes between 8 and 12 residues in length that aligned with Aichi/1968 or Texas/2012 (for human NP) or with swine/Wisconsin/1957 or swine/Indiana/2012 (for swine NP) with no more than one mismatch. Redundant epitopes for the same MHC allele were removed. The epitopes per site are listed in Table S1 and Table S2. See http://jbloom.github.io/epitopefinder/example_NP_CTL_epitopes_H3N2_and_swine.html for code, input data, and detailed documentation.

Figure 3A shows the number of characterized epitopes that contain each site in NP. As can be seen from this figure, the distribution of CTL epitopes is non-uniform along NP's sequence, with some sites falling in many known epitopes and others falling in none. The distributions of epitopes along the NP sequence are highly similar for the human and swine NP homologs. Figure 3B shows the distribution of number of epitopes per site for the human and swine NP homologs. These distributions are nearly indistinguishable (see the Figure 3 legend for statistical testing). Overall, Figure 3 indicates that the human and swine NP homologs contain nearly identical numbers of known human CTL epitopes.

### Human NP exhibits increased evolution in CTL epitopes relative to swine NP

If the NP from human influenza is under selection from human CTLs, we might expect this to lead to an increased rate of fixation of mutations in CTL epitopes. No such selection is expected to occur for the NP from swine influenza, as swine influenza is definitely not under pressure from human CTLs, and is probably not under strong selection even from swine CTLs for the reasons discussed in the Introduction.

**Figure 4:**
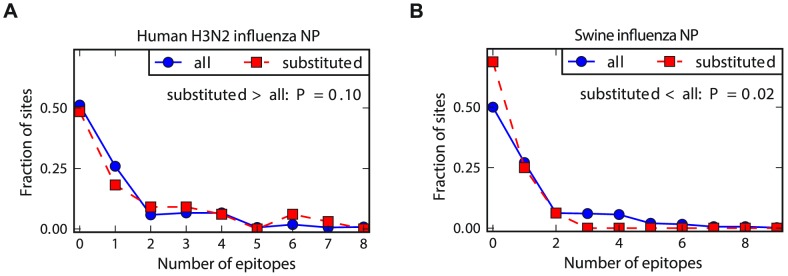
Human NP exhibits increased evolution in CTL epitopes relative to swine NP. The number of CTL epitopes per site for all sites in NP versus those that substituted along the evolutionary trajectories for (A) human and (B) swine influenza. In human influenza, the substituted sites contain more epitopes than average sites – but in swine influenza, the substituted sites contribute to fewer epitopes than average sites. The P-values on the plots are the fraction of random subsets of all sites that contain as many (human NP) or as few (swine NP) total epitopes as the sites that actually substituted during the natural evolution of that homolog. The hypothesis of greatest interest is whether the substituted sites in the human NP contain more epitopes than do substituted sites in the swine NP. To test this hypothesis, we drew paired random subsets of sites from the human and swine NP homolog of the same size as the actual numbers of substituted sites for each homolog, and determined the fraction of these paired random subsets in which the number of epitopes for the human NP exceeded that for the swine NP by at least as much as for the actual data. This test gives a P-value of 0.008, supporting the hypothesis that human NP exhibits an increased rate of evolution in epitopes relative to swine NP. See http://jbloom.github.io/epitopefinder/example_NP_CTL_epitopes_H3N2_and_swine.html for code, input data, and detailed documentation.

To compare the relative rate of substitution in known CTL epitopes for the two NP homologs, we determined the number of epitopes at the sites of the mutations that fixed along the evolutionary trajectories from Figure 2. As shown in Figure 4, for the human NP homolog, the typical fixed mutation falls in more epitopes than an average site – whereas for the swine NP homolog, the typical fixed mutation falls in fewer epitopes than an average site. We interpret these results as follows: the known epitopes in NP tend to involve sites that are less inherently mutationally tolerant than the average site, either due to a tendency of CTLs to target conserved regions or a bias towards the experimental discovery of epitopes in conserved regions of NP (the tendency of characterized CTL epitopes to fall in conserved regions of viral proteins has also been noted by others [60], [61]). This tendency for the epitopes to fall in less mutationally tolerant regions of NP means that in the absence of CTL selection, the site of the typical fixed mutation contributes to fewer epitopes than an average site – this is the case for the swine NP homolog. But for the human NP homolog, selection for adaptive mutations in sites targeted by CTLs is sufficient to cause the fixed mutations to fall in more epitopes than an average site – and in significantly more epitopes than mutations fixed in the swine NP homolog (P = 0.008, see the Figure 4 legend for statistical testing).

### Epistatic interactions are fixed in human but not swine NP

The results in the previous section support the idea that there is pressure for adaptive change in human CTL epitopes for human influenza NP, but not for swine influenza NP. The facts discussed in the Introduction also strongly suggest that swine influenza NP is also under much less selection from swine CTLs than human influenza NP is from human CTLs. How do these differences in adaptive pressures influence the prevalence of epistasis during evolution?

**Figure 5:**
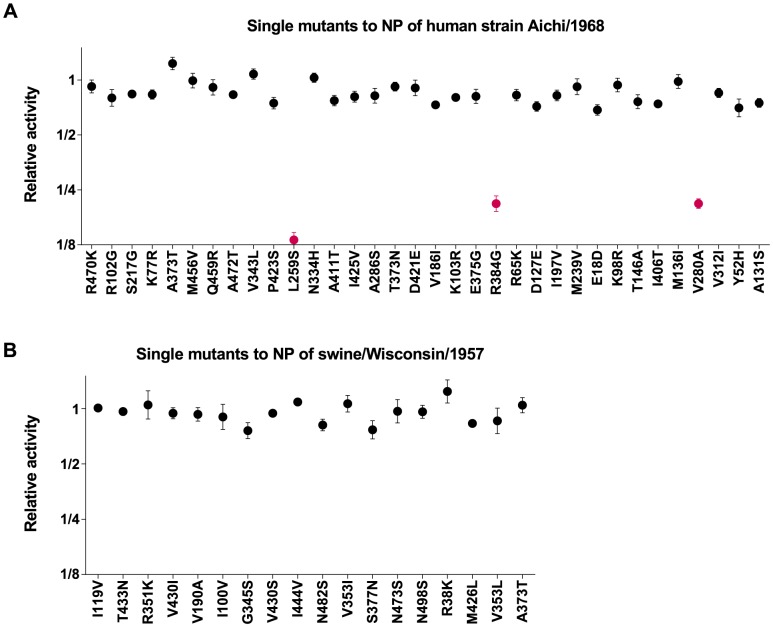
Epistatically constrained mutations are fixed in human but not swine NP. All single mutations that occurred along the evolutionary trajectories were introduced individually into the Aichi/1968 (human NP) or swine/Wisconsin/1957 (swine NP), and the impact of the mutation on the total transcriptional activity of the influenza polymerase was measured experimentally. (A) The effect of the mutations to human NP, as originally reported in [34]. (B) The effect of the mutations to swine NP. Individual mutations that are strongly deleterious are classified as “epistatically constrained,” since their fixation during natural evolution required additional secondary mutations to counteract the deleterious effects. Three epistatically constrained mutations fixed along the human NP trajectory, but no epistatically constrained mutations fixed along the swine NP trajectory. The epistatically constrained mutations are colored red in the plot. The numerical data in Figure 5A are in [34]; the numerical data in Figure 5B are in Table S3.

We have previously performed a systematic test for a specific form of epistasis in the Aichi/1968 human influenza NP [34]. Specifically, we introduced all single mutations from the human NP evolutionary trajectory (Figure 2A) into the initial Aichi/1968 NP parent sequence, and quantified the effect of the mutations on total transcriptional activity by the influenza polymerase in transfected 293T cells. The previously described results from these experiments are shown in Figure 5A. Three of the 34 single mutations are highly deleterious as individual changes to the Aichi/1968 NP, despite the fact that they eventually fixed during the virus's evolution. We have previously shown that these three individually deleterious mutations were able to fix during NP's natural evolution due to epistatic interactions with other mutations that alleviated their deleterious effects [34].

Do similar epistatic interactions occur during the evolution of the swine influenza NP? To experimentally address this question, we introduced all of the single mutations from the swine NP evolutionary trajectory (Figure 2B) into the initial swine/Wisconsin/1957 NP parent sequence, and quantified the effect on transcriptional activity. These results are shown in Figure 5B. None of the mutations have a substantial deleterious effect as individual changes, indicating that none of them were dependent on epistatic interactions with other mutations. Therefore, while the 44-year evolutionary trajectory of the adaptively evolving human influenza NP involved the fixation of three mutations involved in strong epistatic interactions, we see no evidence of similar epistatically interacting substitutions along a 55-year evolutionary trajectory of the swine influenza NP. We acknowledge that the difference in the numbers of substitutions involved in epistatic interactions (3 out of 34 for human influenza NP, 0 out of 18 for swine influenza NP) is not statistically significant, and therefore merely provides anecdotal support for the idea that epistatically interacting substitutions are more common in the adaptively evolving human NP homolog. However, this anecdotal support becomes much more convincing when combined with the observations in the next section.

### Epistasis in human NP occurs at sites enriched in CTL epitopes

**Figure 6:**
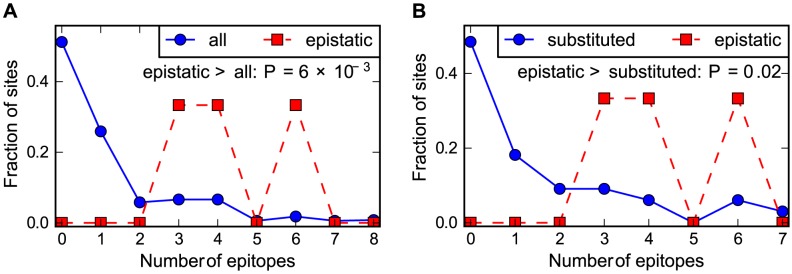
Epistasis in human NP occurs at sites enriched in CTL epitopes. The number of CTL epitopes per site for the sites of the epistatically constrained substitutions in the human influenza NP versus (A) all sites or (B) the full set of sites that substituted along the evolutionary trajectory. The P-values shown on the plots represent the fraction of random subsets that contain as many total epitopes as the actual sites of the epistatically constrained substitutions. See http://jbloom.github.io/epitopefinder/example_NP_CTL_epitopes_H3N2_and_swine.html for code, input data, and detailed documentation.

Is the presence of epistasis in the human but not the swine influenza NP due to the fact that only the former is adaptively evolving to escape from CTL selection? One way to test this idea is to examine whether the epistatic mutations in the human NP are at sites that contribute disproportionately to CTL escape. We have previously noted that the three epistatically constrained mutations in human NP are in known CTL epitopes [34]. Here we use our new comprehensive mapping of CTL epitopes described above to more thoroughly test the hypothesis that epistasis in the human NP is associated with CTL escape. Figure 6 shows that the epistatic mutations occur at sites that contain significantly more CTL epitopes than either average sites in NP or the set of sites that actually substituted along the evolutionary trajectory. Therefore, not only are epistatically interacting substitutions enriched during the evolution of the adaptively evolving human influenza NP relative to its swine influenza homolog – furthermore, the epistasis involves mutations that play an especially important role in the protein's adaptive evolution.

## Discussion

We have used a combination of computational and experimental analyses to examine whether epistasis is more common during adaptive protein evolution. We did this by comparing the evolution of an adaptively evolving NP from human influenza with a closely related homolog from swine influenza that is not under similar pressure for adaptive change. Experimentally, we find that strong epistatic interactions are fixed only during the evolution of the adaptively evolving human influenza NP homolog. Our computational analyses strongly suggest that the different patterns of epistasis are due to the fact that only the human influenza NP homolog is undergoing continuing adaptive evolution. Specifically, mutations that fix in the human influenza NP are significantly more likely to be in sites targeted by human immune memory than are mutations in the swine influenza homolog – and the epistatic interactions all involve sites that are heavily targeted by such immune selection. Overall, these results suggest that epistatically interacting substitutions are significantly enriched in adaptive versus non-adaptive evolution.

Why are epistatically interacting substitutions more prevalent during adaptive evolution? Our experiments probe for epistatic interactions involving a mutation that is individually deleterious but becomes neutral or adaptive when paired with secondary mutations. As discussed in the Introduction, there are two mechanisms by which such epistatic interactions have been shown to fix during adaptive evolution: compensatory mutations and permissive mutations. Our prior work suggests that the epistatic mutations in human influenza NP fix primarily via the latter mechanism, although compensatory mutations may also play a lesser role [34]. Crucially, the driving force for both mechanisms is adaptation. For the compensatory-mutation mechanism, this driving force is obvious: an initial deleterious mutation is more likely to persist long enough to be paired with a compensatory mutation if the initial mutation also confers some adaptive benefit (although mildly deleterious mutations can also fix without compensation, albeit at a lower rate). Somewhat less obviously, a similar force drives the permissive-mutation mechanism: although the initial permissive change is stochastic, the fixation of its subsequent pairing with the mutation that it permits is more likely if the latter change is adaptive [21]. Although epistatically interacting mutations can fix during non-adaptive evolution by similar temporal mechanisms, there is no underlying force to favor these relatively rare epistatic combinations over more abundant and easily accessible non-epistatic mutations.

This explanation can be stated more succinctly in terms specific to the NP homologs studied here. In the absence of adaptation, evolution tends to fix easily accessible non-epistatic mutations that have no adverse effect – in other words, the evolution of the swine influenza NP is dominated by stabilizing selection for retention of function. The human influenza NP is also under strong stabilizing selection for retention of function, but in addition experiences diversifying selection for change in immune epitopes. Some of these adaptive immune-escape mutations have adverse effects on NP function, and so selection biases evolution towards epistatic combinations that enable the adaptive mutations to fix while retaining NP function.

Most experimental studies of epistasis have focused on its role in constraining adaptation [1]–[10]. Our results suggest that caution may be warranted in extrapolating findings about the frequency of epistatically interacting substitutions during adaptation to more general evolutionary scenarios, since such substitutions appear to be more common during adaptive than non-adaptive evolution.

## Materials and Methods

### Phylogenetic tree and mutational paths

The input sequences for construction of the phylogenetic tree (Figure 1) and mutational paths (Figure 2) were downloaded from the Influenza Virus Resource [62]. For human influenza, up to 5 sequences per year were retained from the following lineages: H1N1 (isolation dates from 1918 to 1957, and then from 1977 to 2008), H2N2 (isolation dates from 1957 to 1968), and H3N2 (isolation dates from 1968 to 2012). For swine influenza, up to 5 sequences per year and subtype were retained from North American swine influenza. For the human H1N1 isolated in 1977 or later, 24 years were subtracted from the isolation dates because these sequences are from an influenza lineage revived after being frozen for roughly 24 years [45]. We excluded sequences that were classified as mis-annotated by [63] or that are strong outliers from the molecular clock based on an analysis with RAxML [64] and Path-O-Gen (http://tree.bio.ed.ac.uk/software/pathogen/).

The sequences were translated, date-stamped, and used as input to BEAST [65] with a strict molecular clock, a JTT [66] model of substitution, and a relatively loose coalescent-based prior on the tree. Figure 1 shows a maximum clade credibility tree rendered with FigTree (http://tree.bio.ed.ac.uk/software/figtree/).

The mutational paths in Figure 2 were constructed using the approach described in [34], and were rendered using GraphViz (http://www.graphviz.org/).

The source code, input data, and detailed documentation for the construction of the phylogenetic tree and the mutational paths can be accessed on GitHub via http://jbloom.github.io/mutpath/example_influenza_NP_1918_Descended.html

### Mapping of CTL epitopes

The CTL epitopes were identified by downloading from the Immune Epitope Database [58] all epitopes with a positive T-cell response with source organism *Influenza A virus* and host *Homo sapiens*. We created a new software package, *epitopefinder* (https://github.com/jbloom/epitopefinder), to map specific epitopes to NP.

This mapping was done by parsing all MHC class I peptide epitopes of 8 to 12 residues, and removing as redundant any epitopes that overlapped by 8 or more residues and were from the same MHC class I allele group (see http://hla.alleles.org/nomenclature/naming.html) or from the same MHC class I supertype [59] if no allele group was specified. For redundant epitopes, the shortest epitope sequence was retained. The non-redundant epitopes were aligned to NP: if they aligned to Aichi/1968 or Texas/2012 with no more than one mismatch then they were considered to be present in the human NP homolog, and if they aligned with no more than one mismatch to swine/Wisconsin/1957 or swine/Indiana/2012 with no more than one mismatch then they were considered to be present in the swine NP homolog. The number of epitopes in which each site participates is listed in Tables S1 and S2.

The source code, input data, and detailed documentation for mapping the epitopes and for the computing the P-values can be accessed on GitHub via http://jbloom.github.io/epitopefinder/example_NP_CTL_epitopes_H3N2_and_swine.html

### Experimental assays of NP function

We measured the function of the NP mutants by using flow cytometry to quantify the mean fluorescent intensity of 293T cells 20 hours after they had been transfected with plasmids encoding the NP variant in question, the three influenza polymerase proteins (PB2, PB1, PA), and the fluorescent reporter pHH-PB1flank-eGFP [67]. The data for the human NP homolog in Figure 5A were originally described in [34], and are reprinted here.

The data for the swine NP homolog in Figure 5B were generated by following the protocol described in [34] with the following modifications: the polymerase proteins were derived from the A/California/4/2009 swine-origin H1N1 strain, and the measured signal was normalized to that obtained using the wild-type swine/1957 NP. The polymerase plasmids (pHWCA09tc-PB2, pHWCA09tc-PB1, and pHWCA09tc-PA) have been described previously [68], while the insert for the swine/1957 NP plasmid (pHWswine57-NP) was synthesized commercially and cloned into pHW2000 [69]; the viral-RNA sequences for all four plasmids are in Dataset S1. The A/California/4/2009 swine-origin H1N1 polymerase proteins were chosen because the NP of this strain is closely related to NPs from the latter part of the swine influenza trajectory in Figure 1. We verified that the NP plasmid concentration used in [34] gave signal that was near the midpoint of the assay's dynamic range when using this combination of NP and polymerase genes (Figure S1). The data in Figure 5B represent the mean and standard error of at least three independent replicates; numerical values are in Table S3.

## Supporting Information

Figure S1 The experimentally measured transcriptional activity versus the amount of swine/Wisconsin/1957 NP plasmid transfected into the cells. Based on this plot, we chose to perform our assays using 50 ng of NP plasmid as this concentration is near the middle of the assay's dynamic range. An analogous plot for Aichi/1968 NP has been previously reported as Figure 3—figure supplement 1 of [34].
(EPS)

Table S1 The number of human CTL epitopes per site for the human H3N2 NPs. The number of unique epitopes in which each site participates is listed in CSV format. See http://jbloom.github.io/epitopefinder/example_NP_CTL_epitopes_H3N2_and_swine.html for code, input data, and detailed documentation.
(CSV)

Table S2 The number of human CTL epitopes per site for the swine NPs. The number of unique epitopes in which each site participates is listed in CSV format. See http://jbloom.github.io/epitopefinder/example_NP_CTL_epitopes_H3N2_and_swine.html for code, input data, and detailed documentation.
(CSV)

Table S3 Mean and standard error of the transcriptional activities for the swine NP mutants.
(CSV)

Dataset S1 The viral RNA sequences (reverse complemented) inserted between the RNA polymerase I promoter and terminator in the reverse-genetics plasmids.
(TXT)
